# Supplementary material for: Mechanically robust and personalized silk fibroin-magnesium composite scaffolds with water-responsive shape-memory for irregular bone regeneration
Source: Nat Commun. 2024 May 16;15:4160. doi: 10.1038/s41467-024-48417-8 (PMC11099135; doi:10.1038/s41467-024-48417-8)
Supplement: Supplementary file 1 — Supplementary information [file 41467_2024_48417_MOESM1_ESM.docx]

**Supplementary Information**

**Mechanically robust and personalized silk fibroin-magnesium composite scaffolds with water-responsive shape-memory for irregular bone regeneration**

Zhinan Mao^1,2#^, Xuewei Bi^1,2#^, Chunhao Yu^2^, Lei Chen^3^, Jie Shen^1^, Yongcan Huang^1^, Zihong Wu^5^, Hui Qi^3^, Juan Guan^4^, Xiong Shu^3*^, Binsheng Yu^1*^, Yufeng Zheng^2*^

Z.N. Mao, X.W. Bi, J. Shen, Y.C. Huang, B.S. Yu

^1^Shenzhen Engineering Laboratory of Orthopaedic Regenerative Technologies, Department of Spine Surgery, Peking University Shenzhen Hospital, Shenzhen Peking University-The Hong Kong University of Science and Technology Medical Center,Guangdong province,China.

E-mail: hpyubinsheng@hotmail.com

Z.N. Mao, X.W. Bi, C.H. Yu, Y.F. Zheng

^2^ School of Materials Science and Engineering, Peking University, Beijing, 100871, China.

E-mail: [yfzheng@pku.edu.cn](mailto:yfzheng@pku.edu.cn)

L. Chen, X. Shu, H. Qi

^3^Beijing Research Institute of Orthopedics and Traumatology, Beijing Jishuitan Hospital, Capital Medical University, Beijing, 100035, China.

E-mail: shuxiong@jst-hosp.com.cn

J. Guan

^4^International Research Center for Advanced Structural and Biomaterials, School of Materials Science & Engineering, Beihang University, Beijing 100191, China.

Z.H. Wu

^5^Technical University of Munich, TUM School of Life Sciences, Maximus-von-Imhof-Forum 2, D-85354 Freising, Germany

^#^Zhinan Mao and Xuewei Bi contributed equally to this work.

^*^Corresponding author.

Email: yfzheng@pku.edu.cn (Y.F. Zheng); shuxiong@jst-hosp.com.cn (X. Shu); hpyubinsheng@hotmail.com (B.S. Yu)


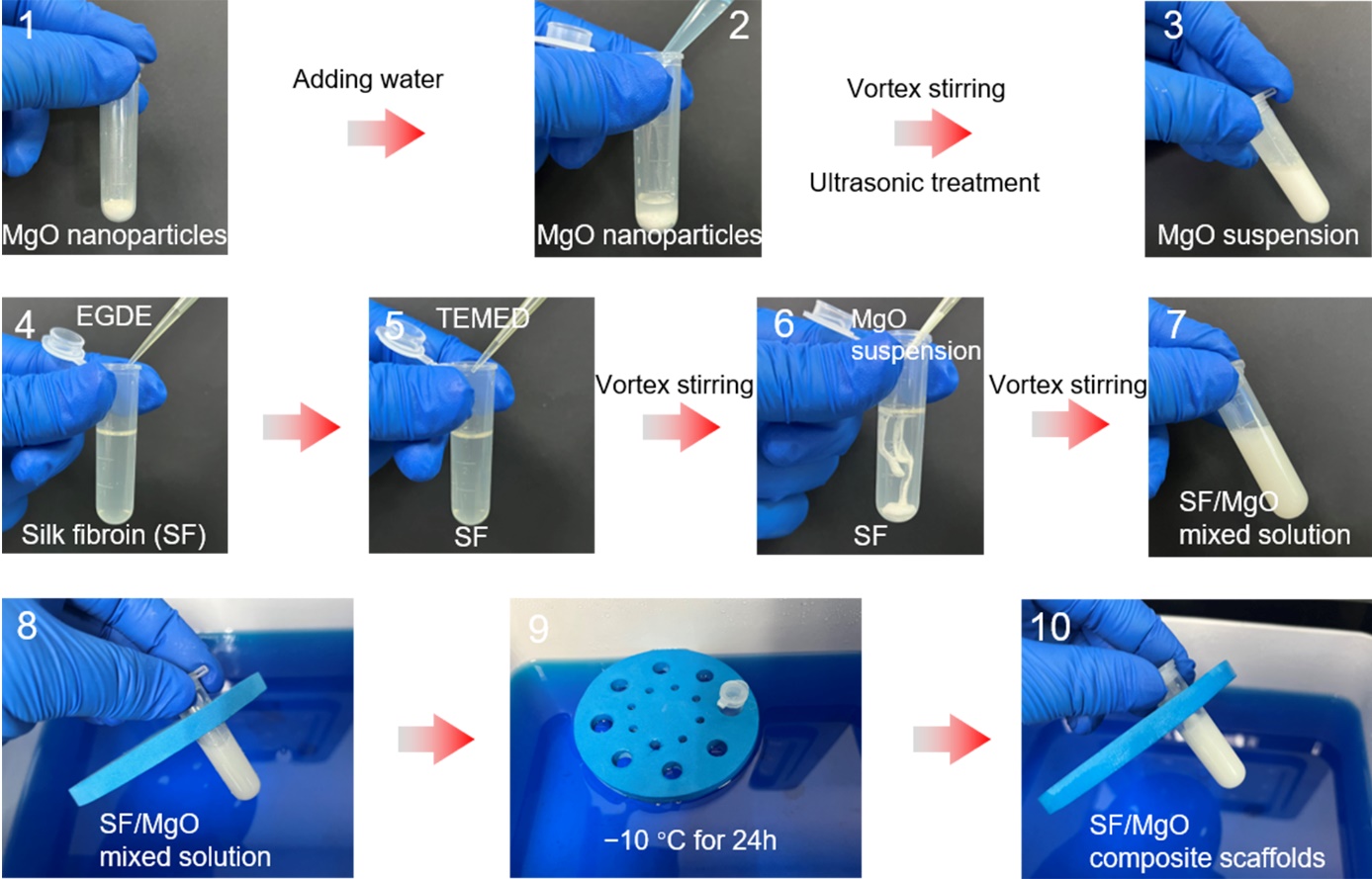


**Supplementary Figure 1**. Preparation process of the SF/MgO composite scaffolds. The SF solution (60 mg/ml) was mixed with aqueous ethylene glycol diglycidyl ether (EGDE, 2 mmol/g) and N,N,N',N'-Tetramethylethylenediamine (TEMED, 0.25 v/v%). Then, nano-MgO particles with different contents (0 wt%, 10 wt%, and 30 wt%, relative to the weight of the SF solute) were added to the SF solution, and the SF/MgO (SF, SF-1nMgO, SF-3nMgO) mixture solution was prepared. After 24 h of cryogelation (-10 °C), the SF/MgO composite scaffolds were constructed.


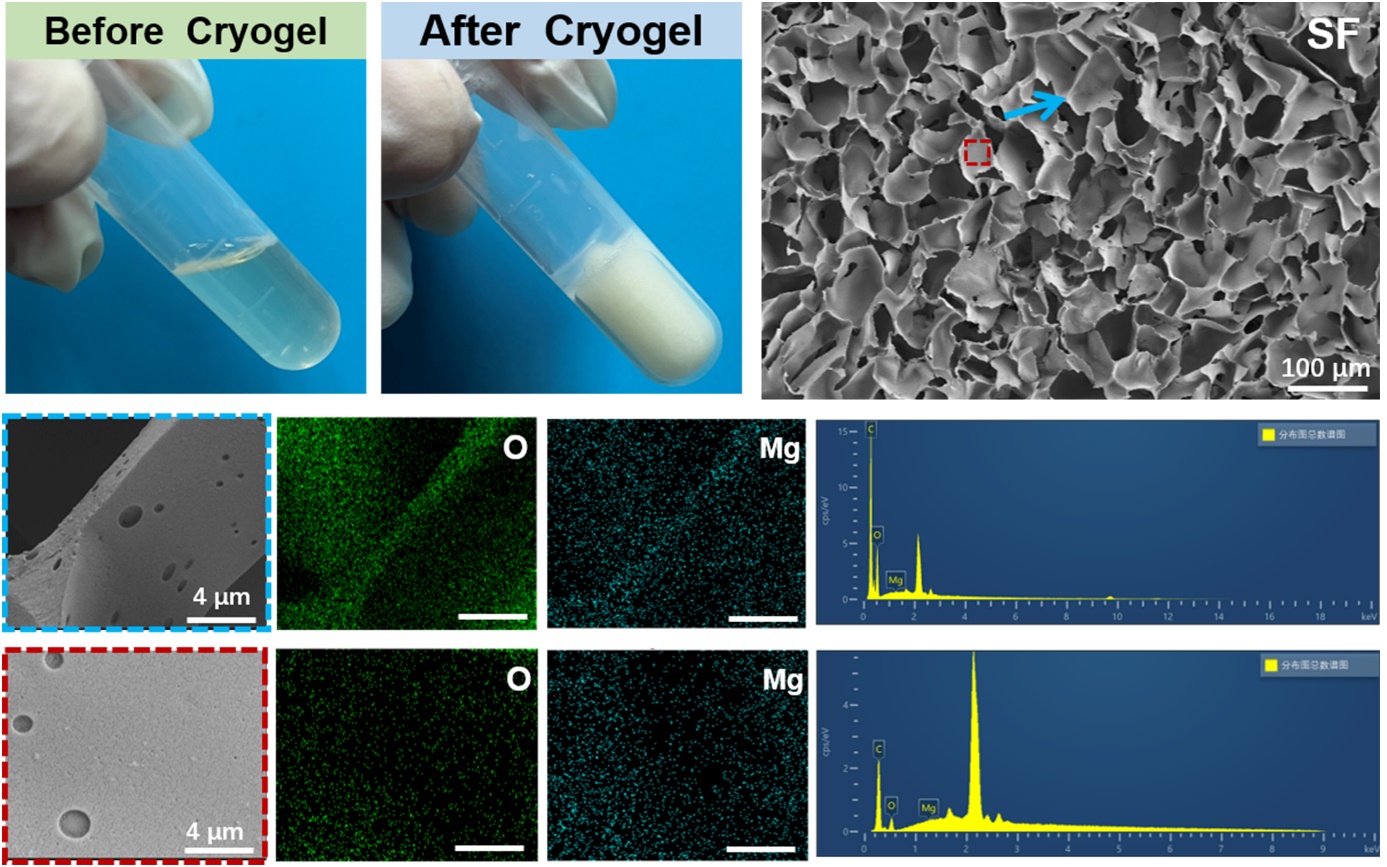


**Supplementary Figure 2**. Morphology and characterisation of SF scaffolds. Including macro-morphologies of SF solutions before and after cryogelation; SEM images of SF scaffolds cross-sections; energy-dispersive spectrometry maps and spectra of the SF scaffolds.


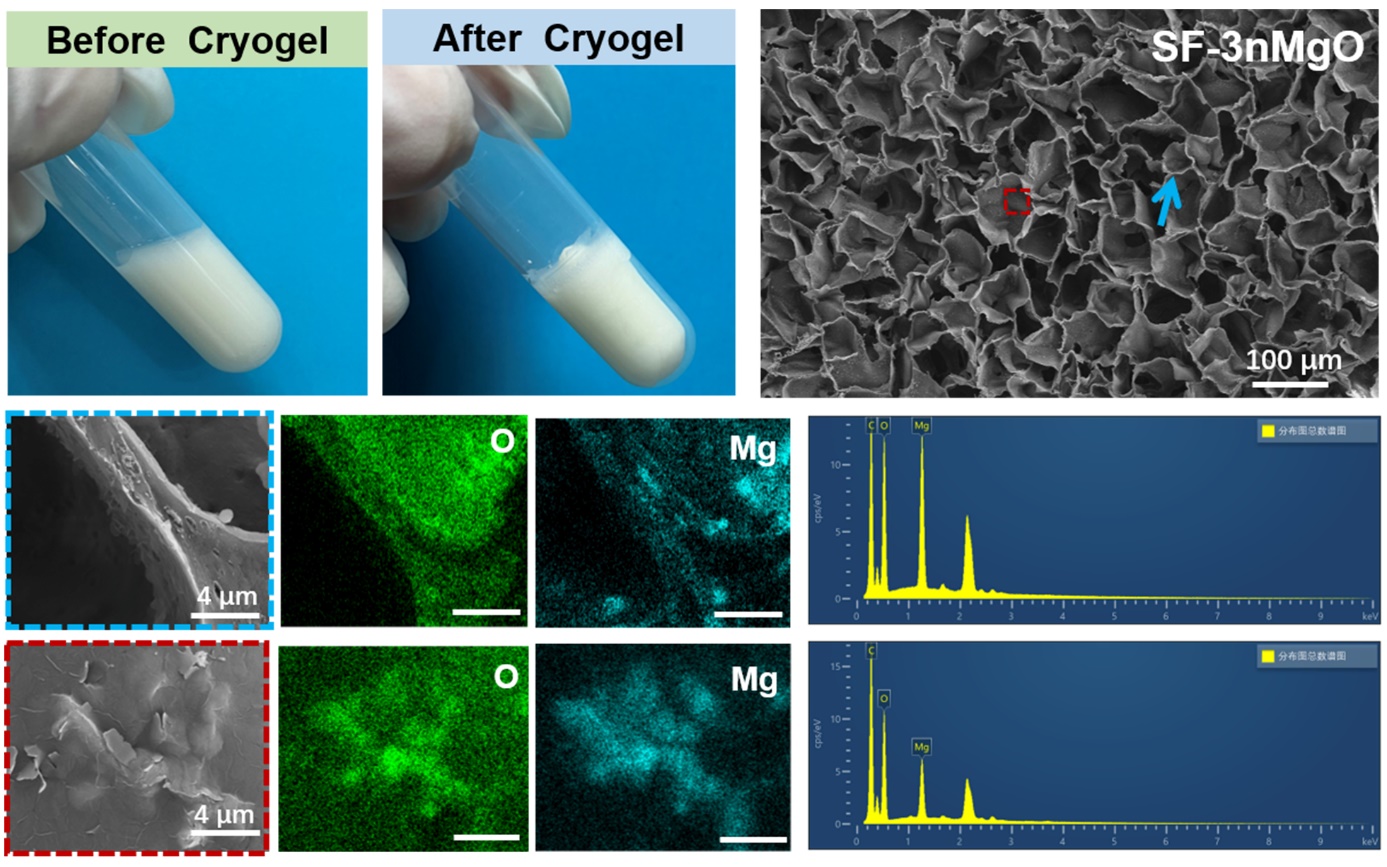


**Supplementary Figure 3**. Morphology and characterization of SF-3nMgO scaffolds. Including macro-morphologies of SF-3nMgO solutions before and after cryogelation; Scanning electron microscopy (SEM) images of SF-3nMgO scaffolds cross-sections; Energy-dispersive spectrometry maps and spectra of the SF scaffolds.


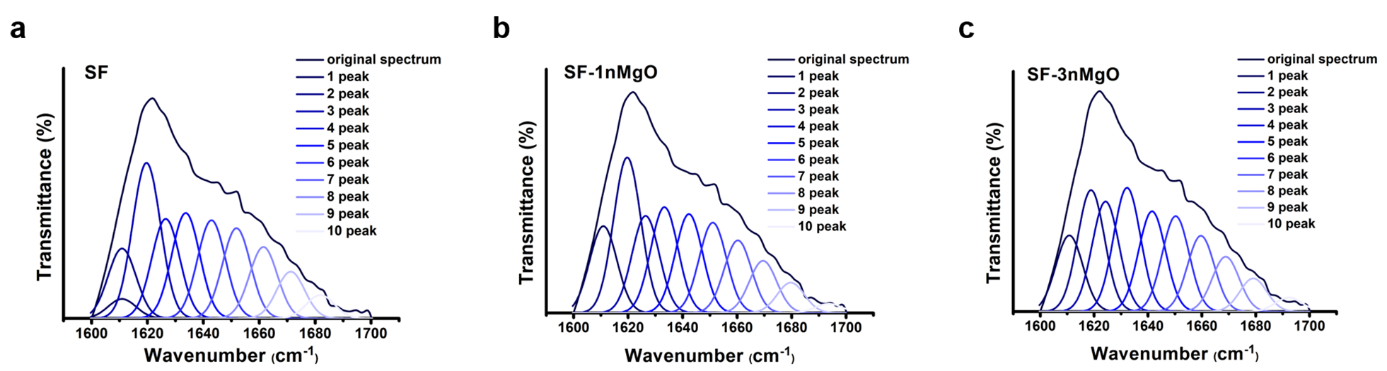


**Supplementary Figure 4**. Representative FTIR spectra after peak fitting. (a) SF scaffolds. (b) SF-1nMgO scaffolds. (c) SF-3nMgO scaffolds. Source data are provided as a Source data file. (*n* = 3 independent samples).


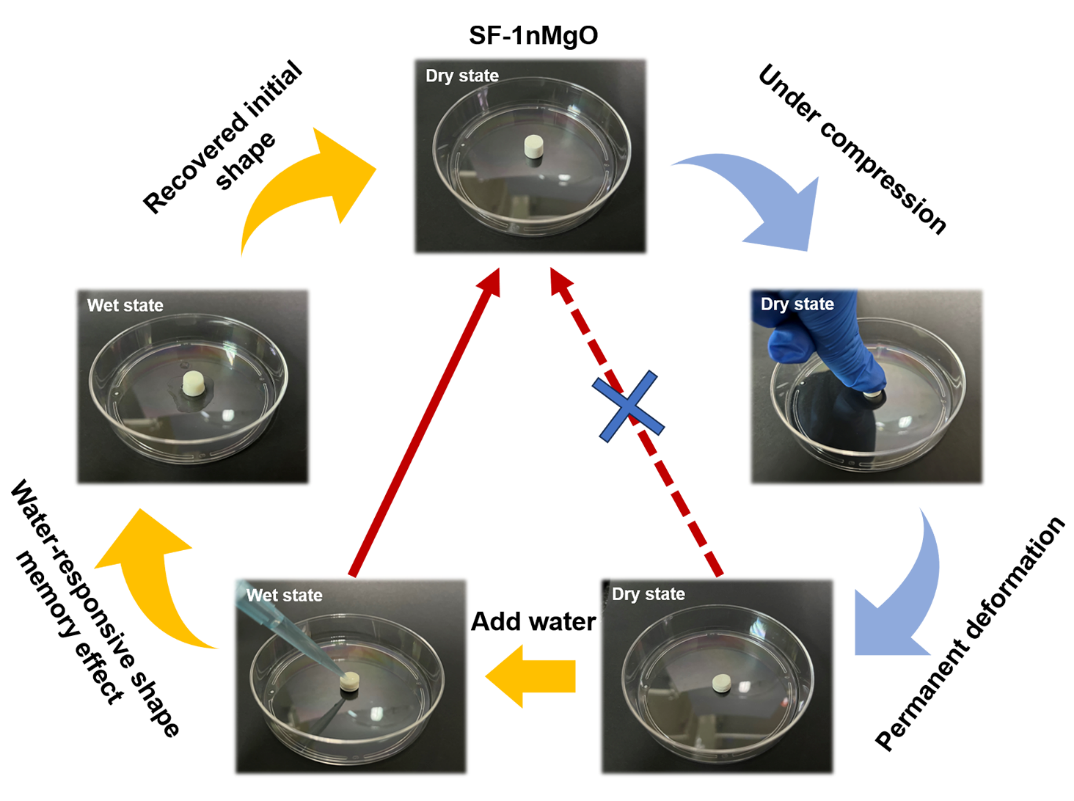


**Supplementary Figure 5**. Schematic illustration of water-responsive shape-memory effect of SF-1nMgO scaffolds. The SF-1nMgO scaffolds in the dry state were permanently deformed after being subjected to external force and had no shape-recovery ability. However, the deformed dry scaffolds that were in contact with water/blood gradually restored their original shape.


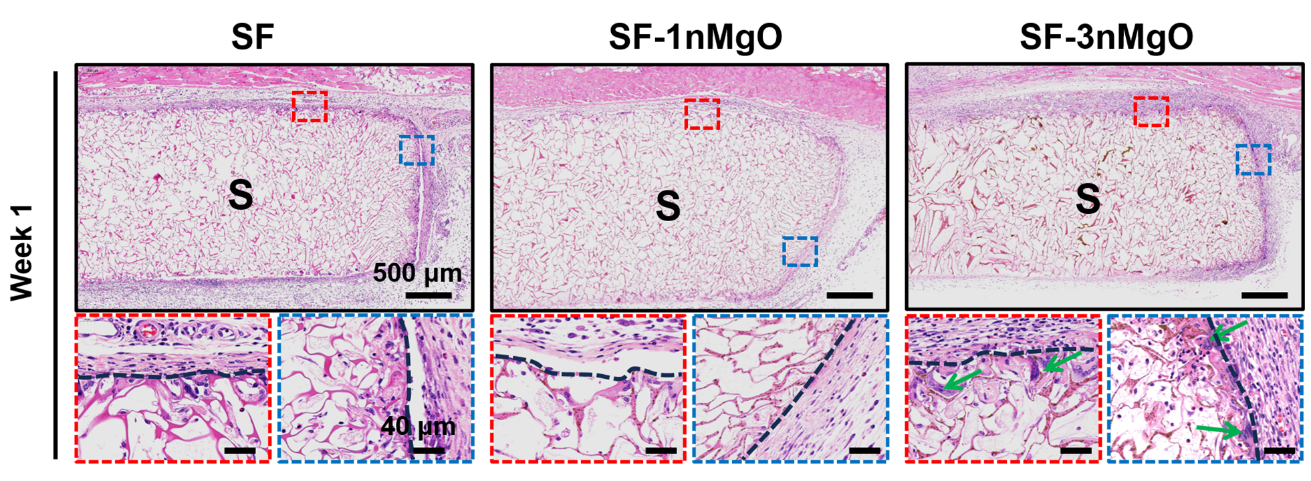


**Supplementary Figure 6**. In vivo histological assessment of subcutaneous implantation of SF, SF-1nMgO and SF-3nMgO scaffolds. Representative images of the H&E staining of different SF scaffolds after 1 week of subcutaneous implantation; scaffolds are labelled with “S”. *n* = 4 biologically independent replicates. Source data are provided as a source data file.


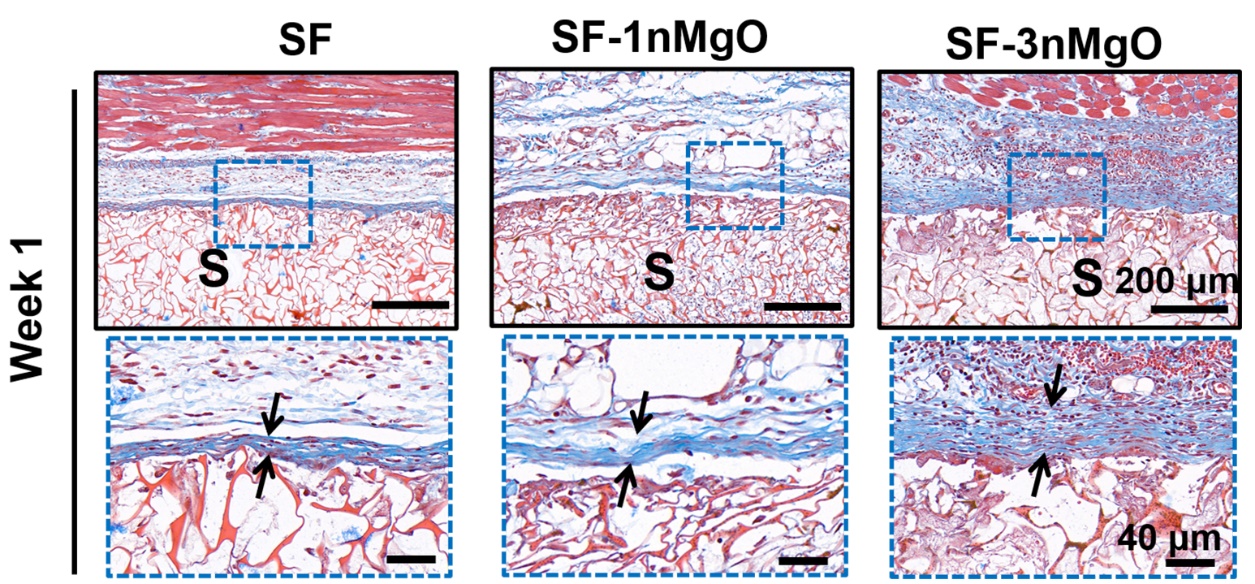


**Supplementary Figure 7**. In vivo histological assessment of subcutaneous implantation to of SF, SF-1nMgO and SF-3nMgO scaffolds. Representative images of the Masson's Trichrome staining of different SF scaffolds after 1 week of subcutaneous implantation; scaffolds are labeled with “S”. *n* = 4 biologically independent replicates. Source data are provided as a source data file.

**Supplementary Table 1.** Primer sequences used for quantitative RT-PCR

| Target Name | Primer | |
| --- | --- | --- |
| GAPDH |  | F-5’-CTGGAGAAACCTGCCAAGTATG-3’ |
|  |  | R-5’-GGTGGAAGAATGGGAGTTGCT-3’ |
| RUNX2 |  | F-5’-GGAACCAAGAAGGCACAGACAG-3’ |
|  |  | R-5’-TGTCTGCCTGGGATCTGTAATCT-3’ |
| OPN |  | F-5’-GATGAACAGTATCCCGATGCCA-3’ |
|  |  | R-5’-GTCTTCCCGTTGCTGTCCTGA-3’ |
| OCN |  | F-5’-TGACAAAGCCTTCATGTCCAA-3’ |
|  |  | R-5’-CTCCAAGTCCATTGTTGAGGTAG-3’ |
| COL1 |  | F-5’-AGAGGCATAAAGGGTCATCGTG-3’ |
|  |  | R-5’-AGACCGTTGAGTCCATCTTTGC-3’ |
