# Supplementary material for: Mechanically robust and personalized silk fibroin-magnesium composite scaffolds with water-responsive shape-memory for irregular bone regeneration
Source: Nat Commun. 2024 May 16;15:4160. doi: 10.1038/s41467-024-48417-8 (PMC11099135; doi:10.1038/s41467-024-48417-8)
Supplement: Supplementary file 2 — Description of Additional Supplementary Files [file 41467_2024_48417_MOESM2_ESM.pdf]

Description of Supplementary Movies

File Name: Supplementary Movie 1

Description: The repeating compression-release tests determined that the SF/MgO composite scaffolds were able to undergo multiple loading and unloading cycles with fast shape recovery, demonstrating an excellent shape-memory and fatigue-resistant ability.

File Name: Supplementary Movie 2

Description: As the applied loads were released, the deformed SF/MgO composite scaffolds could completely recover their original shape without structural failure through water adsorption.

File Name: Supplementary Movie 3

Description: During surgery, the large SF/MgO composite scaffolds could be trimmed according to demand and implanted into defects with small calibre gaps. The shape-memory property enabled the SF/MgO composite scaffolds to instantly restore their original state and then match the shape of the defect, achieving close integration with the bone defect.

File Name: Supplementary Movie 4

Description: The deformed SF/MgO scaffolds had the ability to fully recover the original shape within one second when in contact with water.

File Name: Supplementary Movie 5

Description: The deformed SF/MgO scaffolds had the ability to fully recover the original shape within ten seconds when in contact with blood.

File Name: Supplementary Movie 6

Description: The SF/MgO scaffolds in the wet state or with a certain water content will be deformed under the action of external force, and the scaffolds have a certain shape recovery ability after external force unloading. Both in the dry and wet state, the deformed scaffolds have the ability to fully recover the original shape only when in contact with water.

File Name: Supplementary Movie 7

Description: The dry SF/MgO scaffolds were permanently deformed after being subjected to external force. This phenomenon provided evidence that the dry SF/MgO scaffolds had no shape-recovery ability at 37 °C.
