# Supplementary material for: Mechanically robust and personalized silk fibroin-magnesium composite scaffolds with water-responsive shape-memory for irregular bone regeneration
Source: Nat Commun. 2024 May 16;15:4160. doi: 10.1038/s41467-024-48417-8 (PMC11099135; doi:10.1038/s41467-024-48417-8)
Supplement: Supplementary file 3 — Reporting Summary [file 41467_2024_48417_MOESM3_ESM.pdf]

nature portfolio

Corresponding author(s): Yufeng Zheng

Last updated by author(s): Apr 18, 2024

Reporting Summary

Nature Portfolio wishes to improve the reproducibility of the work that we publish. This form provides structure for consistency and transparency in reporting. For further information on Nature Portfolio policies, see our Editorial Policies and the Editorial Policy Checklist.

Statistics

For all statistical analyses, confirm that the following items are present in the figure legend, table legend, main text, or Methods section.

n/a	Confirmed	
☐	☒	The exact sample size (*n*) for each experimental group/condition, given as a discrete number and unit of measurement
☐	☒	A statement on whether measurements were taken from distinct samples or whether the same sample was measured repeatedly
☐	☒	The statistical test(s) used AND whether they are one- or two-sided<br>*Only common tests should be described solely by name; describe more complex techniques in the Methods section.*
☒	☐	A description of all covariates tested
☒	☐	A description of any assumptions or corrections, such as tests of normality and adjustment for multiple comparisons
☐	☒	A full description of the statistical parameters including central tendency (e.g. means) or other basic estimates (e.g. regression coefficient) AND variation (e.g. standard deviation) or associated estimates of uncertainty (e.g. confidence intervals)
☐	☒	For null hypothesis testing, the test statistic (e.g. *F*, *t*, *r*) with confidence intervals, effect sizes, degrees of freedom and *P* value noted<br>*Give P values as exact values whenever suitable.*
☒	☐	For Bayesian analysis, information on the choice of priors and Markov chain Monte Carlo settings
☒	☐	For hierarchical and complex designs, identification of the appropriate level for tests and full reporting of outcomes
☒	☐	Estimates of effect sizes (e.g. Cohen's *d*, Pearson's *r*), indicating how they were calculated

Our web collection on statistics for biologists contains articles on many of the points above.

Software and code

Policy information about availability of computer code

Data collection	No software was used
Data analysis	Origin 8.5 (Originlab, Northampton MA, USA), IBM SPSS statistics 20.0 (USA), Image-Pro Plus 6.0 and CaseViewer 2.4.

For manuscripts utilizing custom algorithms or software that are central to the research but not yet described in published literature, software must be made available to editors and reviewers. We strongly encourage code deposition in a community repository (e.g. GitHub). See the Nature Portfolio guidelines for submitting code & software for further information.

Data

Policy information about availability of data

All manuscripts must include a data availability statement. This statement should provide the following information, where applicable:

- Accession codes, unique identifiers, or web links for publicly available datasets
- A description of any restrictions on data availability
- For clinical datasets or third party data, please ensure that the statement adheres to our policy

All relevant data that support the findings of this study are available within the article and Supplementary Information. All data are available from the corresponding authors upon request. Source data are provided with this paper.

Research involving human participants, their data, or biological material

Policy information about studies with human participants or human data. See also policy information about sex, gender (identity/presentation), and sexual orientation and race, ethnicity and racism.

Reporting on sex and gender	We don't have related issues since this module is not applicable to my study.
Reporting on race, ethnicity, or other socially relevant groupings	We don't have related issues since this module is not applicable to my study.
Population characteristics	We don't have related issues since this module is not applicable to my study.
Recruitment	We don't have related issues since this module is not applicable to my study.
Ethics oversight	We don't have related issues since this module is not applicable to my study.

Note that full information on the approval of the study protocol must also be provided in the manuscript.

Field-specific reporting

Please select the one below that is the best fit for your research. If you are not sure, read the appropriate sections before making your selection.

☒ Life sciences ☐ Behavioural & social sciences ☐ Ecological, evolutionary & environmental sciences

For a reference copy of the document with all sections, see nature.com/documents/nr-reporting-summary-flat.pdf

Life sciences study design

All studies must disclose on these points even when the disclosure is negative.

Sample size	The samples for scanning electron microscopy were cut to 10 mm × 2 mm (diameter × height). (This is the optimal size allowed for SEM imaging considering operability and integrity of the sample).<br>The samples for X-ray diffraction / Fourier-transform infrared spectroscopy were cut to 10 mm × 2 mm (diameter × height). The slices were then pressed to make them compact for tests. (This is the optimal size of sample for experiment considering the scanning range of the equipment)<br>The dry scaffolds were prepared in 5 mL centrifuge tube using 1 ml of homogeneous reaction solution by cryogel reaction and used as samples for degradation and immersion test experiments. (This is the optimal size of sample for degradation and immersion test experiments considering operability of the sample).<br>The samples for extract liquid were cut to 10 mm × 2 mm (diameter × height). The extract liquid of the scaffolds was prepared according to ISO 10993-12.<br>The compression testing samples were cut to 10 mm × 10 mm (diameter × height). For compression test, the height of the sample is guaranteed to be more than 1 time of the diameter. (This is also the optimal size of sample considering fixture size and load capacity of the equipment)<br>The scaffolds with a cylindrical shape (1 cm diameter, 2 mm height) were used for subcutaneous implantation. (This is the optimal size of implant for subcutaneous implantation considering operability of the sample)<br>The scaffolds with a cylindrical shape (4 mm diameter, 1 mm height) were used for rat skull defect model. Because the thickness of the rat skull is about 1 mm, the 4mm diameter defect is a critical bone defect.(This is the optimal size of implant for skull defect model)
Data exclusions	Data were not excluded from analysis.
Replication	All the quantitative data in this study has been repeated for as least 3 times independently. The n numbers are presented in each figure legend. All attempts at replication were shown with scatter in related figures.
Randomization	The samples in this experiment were randomly assigned, and there was no considered control.
Blinding	The data used for statistics in the article were measured in the same environment and there is no blinding issue.

Behavioural & social sciences study design

All studies must disclose on these points even when the disclosure is negative.

Study description	*Briefly describe the study type including whether data are quantitative, qualitative, or mixed-methods (e.g. qualitative cross-sectional, quantitative experimental, mixed-methods case study).*
Research sample	*State the research sample (e.g. Harvard university undergraduates, villagers in rural India) and provide relevant demographic information (e.g. age, sex) and indicate whether the sample is representative. Provide a rationale for the study sample chosen. For*

	studies involving existing datasets, please describe the dataset and source.
Sampling strategy	*Describe the sampling procedure (e.g. random, snowball, stratified, convenience). Describe the statistical methods that were used to predetermine sample size OR if no sample-size calculation was performed, describe how sample sizes were chosen and provide a rationale for why these sample sizes are sufficient. For qualitative data, please indicate whether data saturation was considered, and what criteria were used to decide that no further sampling was needed.*
Data collection	*Provide details about the data collection procedure, including the instruments or devices used to record the data (e.g. pen and paper, computer, eye tracker, video or audio equipment) whether anyone was present besides the participant(s) and the researcher, and whether the researcher was blind to experimental condition and/or the study hypothesis during data collection.*
Timing	*Indicate the start and stop dates of data collection. If there is a gap between collection periods, state the dates for each sample cohort.*
Data exclusions	*If no data were excluded from the analyses, state so OR if data were excluded, provide the exact number of exclusions and the rationale behind them, indicating whether exclusion criteria were pre-established.*
Non-participation	*State how many participants dropped out/declined participation and the reason(s) given OR provide response rate OR state that no participants dropped out/declined participation.*
Randomization	*If participants were not allocated into experimental groups, state so OR describe how participants were allocated to groups, and if allocation was not random, describe how covariates were controlled.*

Ecological, evolutionary & environmental sciences study design

All studies must disclose on these points even when the disclosure is negative.

Study description	Here, a dual-selective polymer-metal thermal emitter was developed, which exhibited selective emission in both atmospheric transparent windows (8–13 µm and 16–25 µm) and reflection in the remaining mid-infrared and solar wavebands. As a result, the dual-selective thermal emitter exhibited an ultrahigh subambient daytime cooling performance, surpassing existing typical thermal emitters and commercial counterparts.
Research sample	We don't have related issues.
Sampling strategy	We don't have related issues.
Data collection	The data used for statistics in the article were collected by a contact K-type thermocouple pyrometer in the same locations.
Timing and spatial scale	The data used for the statistics were obtained in the same location, specifically a continuous test in a dry environment for 40 min to 24 hours, with data recorded every minute. The test times were sunny outdoor in the Ulan Buh desert, Inner Mongolia, China,(6,7 and 9 September 2022) and in Beijing, China (31 January 2023, 29 March 2023, and 8 October 2023).
Data exclusions	Data were not excluded from analysis
Reproducibility	All thermal measurement results in the article are repeatable when measured under similar outdoor conditions (such as ambient temperature, relative humidity, weather condition, solar irradiation and wind speed).
Randomization	We calculated the results for all samples without randomness.
Blinding	The data used for statistics in the article were measured by calibrated thermocouples in the same environment and there is no blinding issue.

Did the study involve field work? ☐ Yes ☒ No

Field work, collection and transport

Field conditions	Ulan Buh desert, Inner Mongolia, China,(from 6 to 7, September 2022): with a ambient temperature of 15~40 °C, a relative humidity of 5%-50%, and a top solar radiation of ~850 W m-2;<br>Ulan Buh desert, Inner Mongolia, China,(9 September 2022): with a ambient temperature of ~31 °C, a relative humidity of 12%, and a solar radiation of 600~800 W m-2;<br>Beijing, China (31 January 2023): with a ambient temperature of ~15 °C, a relative humidity of 10%, and a solar radiation of 600~800 W m-2;<br>Beijing, China (29 March 2023): with a ambient temperature of ~30 °C, a relative humidity of 13%, and a solar radiation of ~850 W m-2;<br>Beijing, China (8 October 2023): with a ambient temperature of ~30 °C, a relative humidity of 18%, and a solar radiation of 400~600 W m-2;
Location	Ulan Buh desert, Inner Mongolia, China and Beijing, China

Access & import/export	There are no local limitations for accessing the aforementioned locations & importing/exporting our samples
Disturbance	The extra thermal radiation from surrounding buildings can affect the test results and to avoid this effect, an open and unobstructed test site is required

Reporting for specific materials, systems and methods

We require information from authors about some types of materials, experimental systems and methods used in many studies. Here, indicate whether each material, system or method listed is relevant to your study. If you are not sure if a list item applies to your research, read the appropriate section before selecting a response.

Materials & experimental systems

n/a	Involved in the study
☐	☒ Antibodies
☐	☒ Eukaryotic cell lines
☒	☐ Palaeontology and archaeology
☐	☒ Animals and other organisms
☒	☐ Clinical data
☒	☐ Dual use research of concern
☒	☐ Plants

Methods

n/a	Involved in the study
☒	☐ ChIP-seq
☒	☐ Flow cytometry
☒	☐ MRI-based neuroimaging

Antibodies

Antibodies used

Osteocalcin Antibody; Affinity; DF12303; 85n2245; Polyclonal; WB ; IHC ; IF/ICC
Osteopontin Antibody; Affinity; AF0227; 50x3141; Polyclonal; WB ; IHC ; IF/ICC
GAPDH Antibody; Affinity; AF7021; 82u0922 ; Polyclonal; WB ; IHC ; IF/ICC
Collagen I Antibody; Affinity; AF7001; 28u3150; Polyclonal; WB ; IHC ; IF/ICC
CD31 Antibody; Affinity; AF6191; 25u3337; Polyclonal; WB ; IHC ; IF/ICC
VEGF Antibody; Affinity; AF5131; 83m8093; Polyclonal; WB ; IHC ; IF/ICC
BMP2 Antibody; Affinity; AF5163; 15v8605; Polyclonal; WB ; IHC ; IF/ICC
RUNX2 Antibody; Affinity; AF5186; 27c2645; Polyclonal; WB ; IHC ; IF/ICC

Validation

Osteocalcin Antibody:
https://www.affbiotech.cn/goods-15393-DF12303-Osteocalcin_Antibody.html
Osteopontin Antibody:
https://www.affbiotech.cn/goods-143-AF0227-Osteopontin_Antibody.html
GAPDH Antibody:
https://www.affbiotech.com/goods-6289-AF7021-GAPDH_Antibody.html
Collagen I Antibody:
https://www.affbiotech.cn/goods-2057-AF7001-Collagen_I_Antibody.html
CD31 Antibody:
https://www.affbiotech.cn/goods-1822-AF6191-CD31_Antibody.html
VEGFA Antibody:
https://www.affbiotech.cn/goods-4438-AF5131-VEGFA_Antibody.html
BMP2 Antibody:
https://www.affbiotech.cn/goods-4470-AF5163-BMP2_Antibody.html
RUNX2 Antibody:
https://www.affbiotech.cn/goods-4493-AF5186-RUNX2_Antibody.html

Eukaryotic cell lines

Policy information about cell lines and Sex and Gender in Research

Cell line source(s)	Mouse embryo osteoblast precursor (MC3T3-E1) cells (PUMC000012, Cell Resource Center, IBMS, CAMS/PUMC) were utilised for evaluating cell viability, proliferation and migration;<br>Bone mesenchymal stem cells (CP-R131, Procell Life Science & Technology Co,. Ltd) were utilised to evaluate the osteogenic differentiation.
Authentication	Commercially purchased cell lines were not authenticated by study participants.
Mycoplasma contamination	All cell lines were tested negative for mycoplasma contamination.
Commonly misidentified lines (See ICLAC register)	No commonly misidentified cell lines were used in the study.

Palaeontology and Archaeology

Specimen provenance	*Provide provenance information for specimens and describe permits that were obtained for the work (including the name of the issuing authority, the date of issue, and any identifying information). Permits should encompass collection and, where applicable, export.*
Specimen deposition	*Indicate where the specimens have been deposited to permit free access by other researchers.*
Dating methods	*If new dates are provided, describe how they were obtained (e.g. collection, storage, sample pretreatment and measurement), where they were obtained (i.e. lab name), the calibration program and the protocol for quality assurance OR state that no new dates are provided.*

☐ Tick this box to confirm that the raw and calibrated dates are available in the paper or in Supplementary Information.

Ethics oversight	*Identify the organization(s) that approved or provided guidance on the study protocol, OR state that no ethical approval or guidance was required and explain why not.*

Note that full information on the approval of the study protocol must also be provided in the manuscript.

Animals and other research organisms

Policy information about studies involving animals; ARRIVE guidelines recommended for reporting animal research, and Sex and Gender in Research

Laboratory animals	6-week-old male Sprague-Dawley rats weighing 220-230 g were used for in vivo subcutaneous implantation and skull defect model.
Wild animals	No wild animals were used in the study.
Reporting on sex	The male Sprague-Dawley (SD) rats were used for in vivo subcutaneous implantation.<br>There are several reasons why male SD rats are selected as subjects in scientific experiments:<br>1. Physiological differences: Female mammals have oestrous cycles that eventually turn into menstrual periods if they are not pregnant, potentially adding a tricky variable to scientific trials and data analysis. However, a considerable number of studies have also found that female mice do not respond differently to drugs at different times in the physiological cycle.<br>2. Ease of control: Male rats can be more easily controlled and standardized during the experiment because they do not have physiological changes such as the menstrual cycle.<br>3. Avoid confusion: The use of male animals can avoid the influence of physiological state changes such as estrus or pregnancy on the experimental results.
Field-collected samples	No field collected samples were used in the study.
Ethics oversight	All procedures were approved by the animal care and use committee of Beijing Jishuitan Hospital regarding the Guiding Principles for the care and use of experimental animals (Number: 2022-12-03).

Note that full information on the approval of the study protocol must also be provided in the manuscript.

Clinical data

Policy information about clinical studies

All manuscripts should comply with the ICMJEguidelines for publication of clinical research and a completedCONSORT checklist must be included with all submissions.

Clinical trial registration	*Provide the trial registration number from ClinicalTrials.gov or an equivalent agency.*
Study protocol	*Note where the full trial protocol can be accessed OR if not available, explain why.*
Data collection	*Describe the settings and locales of data collection, noting the time periods of recruitment and data collection.*
Outcomes	*Describe how you pre-defined primary and secondary outcome measures and how you assessed these measures.*

Dual use research of concern

Policy information about dual use research of concern

Hazards

Could the accidental, deliberate or reckless misuse of agents or technologies generated in the work, or the application of information presented in the manuscript, pose a threat to:

No | Yes
- [ ] [ ] Public health
- [ ] [ ] National security
- [ ] [ ] Crops and/or livestock
- [ ] [ ] Ecosystems
- [ ] [ ] Any other significant area

Experiments of concern

Does the work involve any of these experiments of concern:

No | Yes
- [ ] [ ] Demonstrate how to render a vaccine ineffective
- [ ] [ ] Confer resistance to therapeutically useful antibiotics or antiviral agents
- [ ] [ ] Enhance the virulence of a pathogen or render a nonpathogen virulent
- [ ] [ ] Increase transmissibility of a pathogen
- [ ] [ ] Alter the host range of a pathogen
- [ ] [ ] Enable evasion of diagnostic/detection modalities
- [ ] [ ] Enable the weaponization of a biological agent or toxin
- [ ] [ ] Any other potentially harmful combination of experiments and agents

Plants

Seed stocks

Report on the source of all seed stocks or other plant material used. If applicable, state the seed stock centre and catalogue number. If plant specimens were collected from the field, describe the collection location, date and sampling procedures.

Novel plant genotypes

Describe the methods by which all novel plant genotypes were produced. This includes those generated by transgenic approaches, gene editing, chemical/radiation-based mutagenesis and hybridization. For transgenic lines, describe the transformation method, the number of independent lines analyzed and the generation upon which experiments were performed. For gene-edited lines, describe the editor used, the endogenous sequence targeted for editing, the targeting guide RNA sequence (if applicable) and how the editor was applied.

Authentication

Describe any authentication procedures for each seed stock used or novel genotype generated. Describe any experiments used to assess the effect of a mutation and, where applicable, how potential secondary effects (e.g. second site T-DNA insertions, mosiacism, off-target gene editing) were examined.

ChIP-seq

Data deposition

- [ ] Confirm that both raw and final processed data have been deposited in a public database such as GEO.
- [ ] Confirm that you have deposited or provided access to graph files (e.g. BED files) for the called peaks.

Data access links
May remain private before publication.

For "Initial submission" or "Revised version" documents, provide reviewer access links. For your "Final submission" document, provide a link to the deposited data.

Files in database submission

Provide a list of all files available in the database submission.

Genome browser session
(e.g. UCSC)

Provide a link to an anonymized genome browser session for "Initial submission" and "Revised version" documents only, to enable peer review. Write "no longer applicable" for "Final submission" documents.

Methodology

Replicates

Describe the experimental replicates, specifying number, type and replicate agreement.

Sequencing depth

Describe the sequencing depth for each experiment, providing the total number of reads, uniquely mapped reads, length of reads and whether they were paired- or single-end.

Antibodies

Describe the antibodies used for the ChIP-seq experiments; as applicable, provide supplier name, catalog number, clone name, and lot number.

Peak calling parameters

Specify the command line program and parameters used for read mapping and peak calling, including the ChIP, control and index files used.

Data quality	*Describe the methods used to ensure data quality in full detail, including how many peaks are at FDR 5% and above 5-fold enrichment.*
Software	*Describe the software used to collect and analyze the ChIP-seq data. For custom code that has been deposited into a community repository, provide accession details.*

Flow Cytometry

Plots

Confirm that:

- [ ] The axis labels state the marker and fluorochrome used (e.g. CD4-FITC).
- [ ] The axis scales are clearly visible. Include numbers along axes only for bottom left plot of group (a 'group' is an analysis of identical markers).
- [ ] All plots are contour plots with outliers or pseudocolor plots.
- [ ] A numerical value for number of cells or percentage (with statistics) is provided.

Methodology

Sample preparation	*Describe the sample preparation, detailing the biological source of the cells and any tissue processing steps used.*
Instrument	*Identify the instrument used for data collection, specifying make and model number.*
Software	*Describe the software used to collect and analyze the flow cytometry data. For custom code that has been deposited into a community repository, provide accession details.*
Cell population abundance	*Describe the abundance of the relevant cell populations within post-sort fractions, providing details on the purity of the samples and how it was determined.*
Gating strategy	*Describe the gating strategy used for all relevant experiments, specifying the preliminary FSC/SSC gates of the starting cell population, indicating where boundaries between "positive" and "negative" staining cell populations are defined.*

- [ ] Tick this box to confirm that a figure exemplifying the gating strategy is provided in the Supplementary Information.

Magnetic resonance imaging

Experimental design

Design type	*Indicate task or resting state; event-related or block design.*
Design specifications	*Specify the number of blocks, trials or experimental units per session and/or subject, and specify the length of each trial or block (if trials are blocked) and interval between trials.*
Behavioral performance measures	*State number and/or type of variables recorded (e.g. correct button press, response time) and what statistics were used to establish that the subjects were performing the task as expected (e.g. mean, range, and/or standard deviation across subjects).*

Acquisition

Imaging type(s)	*Specify: functional, structural, diffusion, perfusion.*
Field strength	*Specify in Tesla*
Sequence & imaging parameters	*Specify the pulse sequence type (gradient echo, spin echo, etc.), imaging type (EPI, spiral, etc.), field of view, matrix size, slice thickness, orientation and TE/TR/flip angle.*
Area of acquisition	*State whether a whole brain scan was used OR define the area of acquisition, describing how the region was determined.*

Diffusion MRI ☐ Used ☐ Not used

Preprocessing

Preprocessing software	*Provide detail on software version and revision number and on specific parameters (model/functions, brain extraction, segmentation, smoothing kernel size, etc.).*
Normalization	*If data were normalized/standardized, describe the approach(es): specify linear or non-linear and define image types used for transformation OR indicate that data were not normalized and explain rationale for lack of normalization.*

Normalization template	*Describe the template used for normalization/transformation, specifying subject space or group standardized space (e.g. original Talairach, MNI305, ICBM152) OR indicate that the data were not normalized.*
Noise and artifact removal	*Describe your procedure(s) for artifact and structured noise removal, specifying motion parameters, tissue signals and physiological signals (heart rate, respiration).*
Volume censoring	*Define your software and/or method and criteria for volume censoring, and state the extent of such censoring.*

Statistical modeling & inference

Model type and settings	*Specify type (mass univariate, multivariate, RSA, predictive, etc.) and describe essential details of the model at the first and second levels (e.g. fixed, random or mixed effects; drift or auto-correlation).*
Effect(s) tested	*Define precise effect in terms of the task or stimulus conditions instead of psychological concepts and indicate whether ANOVA or factorial designs were used.*

Specify type of analysis: ☐ Whole brain ☐ ROI-based ☐ Both

Statistic type for inference (See Eklund et al. 2016)	*Specify voxel-wise or cluster-wise and report all relevant parameters for cluster-wise methods.*
Correction	*Describe the type of correction and how it is obtained for multiple comparisons (e.g. FWE, FDR, permutation or Monte Carlo).*

Models & analysis

n/a	Involved in the study
☐	☐ Functional and/or effective connectivity
☐	☐ Graph analysis
☐	☐ Multivariate modeling or predictive analysis

Functional and/or effective connectivity	*Report the measures of dependence used and the model details (e.g. Pearson correlation, partial correlation, mutual information).*
Graph analysis	*Report the dependent variable and connectivity measure, specifying weighted graph or binarized graph, subject- or group-level, and the global and/or node summaries used (e.g. clustering coefficient, efficiency, etc.).*
Multivariate modeling and predictive analysis	*Specify independent variables, features extraction and dimension reduction, model, training and evaluation metrics.*
